# Case report: Sympathetic ophthalmia after vitrectomies in a patient with Von Hippel–Lindau syndrome

**DOI:** 10.3389/fmed.2023.1118913

**Published:** 2023-02-13

**Authors:** Xiaonan Zhuang, Fengjuan Gao, Zhongcui Sun, Xinyi Ding, Gezhi Xu

**Affiliations:** ^1^Department of Ophthalmology, Eye and ENT Hospital, Fudan University, Shanghai, China; ^2^Shanghai Key Laboratory of Visual Impairment and Restoration, Fudan University, Shanghai, China; ^3^NHC Key Laboratory of Myopia, Fudan University, Shanghai, China

**Keywords:** sympathetic ophthalmia, autoimmune, Von Hippel–Lindau syndrome, optical coherence tomography angiography, choriocapillaris, choroid

## Abstract

**Background:**

Sympathetic ophthalmia (SO) is a rare but sight-threatening uveitis, and most observations have been made after typical manifestations occur. This report focuses on the choroidal changes detected by multimodal imaging at the presymptomatic stage of SO, which is implicated in the early recognition of SO.

**Case presentation:**

A 21-year-old woman suffered from decreased vision in the right eye and was diagnosed with retinal capillary hemangioblastomas associated with Von Hippel–Lindau syndrome. The patient underwent two 23-G pars plana vitrectomies (PPVs), soon after which typical signs of SO manifested. SO resolved quickly after the oral administration of prednisone and remained stable during the follow-up of more than 1 year. The retrospective analysis revealed preexisting bilaterally increased choroidal thickness, dots of flow void on the choroid, and choriocapillaris en-face slabs in optical coherence tomography angiography (OCTA) after the first PPV, which were all reversed by corticosteroid treatment.

**Conclusion:**

The case report highlights the involvement of the choroid and choriocapillaris at the presymptomatic stage of SO after the first inciting event. Abnormally thickened choroid and flow void dots suggested that SO had started and an ensuing surgery would run the risk of exacerbating SO. OCTA scanning of both eyes should be ordered routinely for patients with a history of trauma or intraocular surgeries, especially before the next surgical intervention. The report also suggests that non-human leukocyte antigen gene variation may also regulate the progression of SO, which requires further laboratory investigations.

## Introduction

Sympathetic ophthalmia (SO) is a bilaterally granulomatous panuveitis that is rare but potentially sight-threatening with high rates of visual loss. The cumulative incidence of SO after inciting events is 0.044% (1). SO develops following penetrating injury or intraocular surgeries, and intraocular surgeries, especially vitreoretinal surgeries, have been identified as the main risk factor for SO (2, 3). Furthermore, multiple surgical interventions have been regarded as important in SO (2). In a recent study, the risk of developing SO rose exponentially with the number of vitreoretinal surgeries, although it was low after a single vitreoretinal procedure (4). Improvements in multimodal imaging techniques help to understand the pathogenesis of SO, aid the early diagnosis of SO, and monitor disease progression and response to therapy (5). However, there have been few follow-ups of imaging changes from the first inciting event to the final diagnosis of SO.

T cell-mediated autoimmune response underlies the pathogenesis of SO, and growing evidence supports the critical role of the CD4+ T cell subset, including Th17 cells in autoimmune uveitis including SO (6, 7). Von Hippel–Lindau (VHL) deficiency halts Th17 differentiation, and thus, is implicated in controlling autoimmune disease (8). Germline mutations leading to *VHL* gene loss of function cause VHL syndrome. It is characterized by capillary hemangioblastomas of the retina and central nervous system, clear cell renal carcinoma, pheochromocytomas, and pancreatic neuroendocrine tumors (9). Genetic background is also pivotal in susceptibility to SO, and the reported genetic loci include the human leukocyte antigen (HLA) genes and PDCD1 (10). However, the effects of germline VHL deficiency on the progression of SO remain unknown.

To the best of our knowledge, this report presents the first case of SO in the context of VHL syndrome and describes the earliest changes in the choroid and choriocapillaris observed by the optical coherence tomography angiography (OCTA), which preceded the clinical symptoms of SO and even retinal changes observed by optical coherence tomography (OCT). The unreported presymptomatic signs of SO after the first inciting event may be helpful in evaluating the risk of aggravating SO by additional surgery.

## Case report

**Figure 1:**
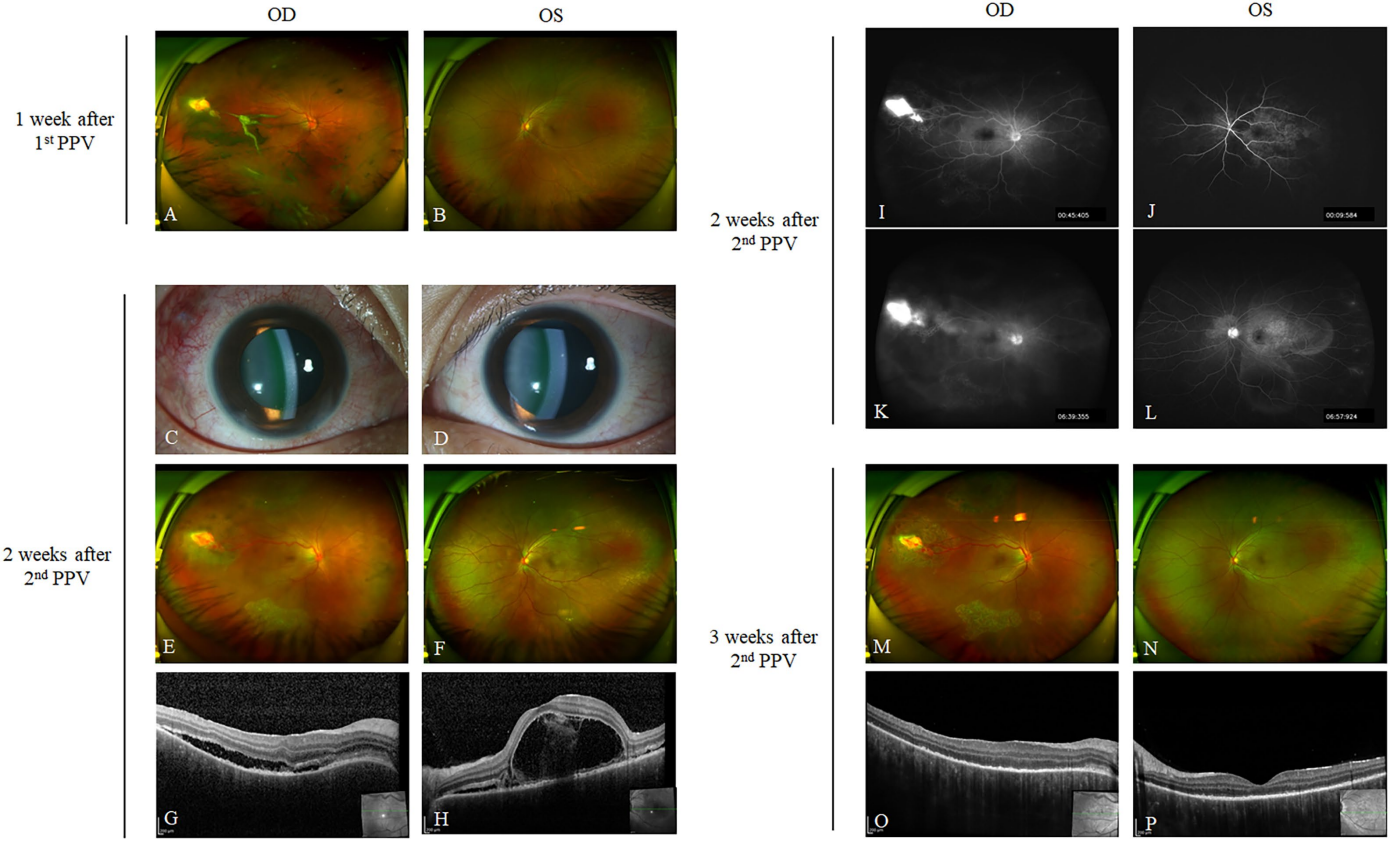
Typical manifestations and resolution of SO. One week after the first PPV, residual vitreous hemorrhage and a yellowish supertemporal lesion were found in the right eye on the initial arrival at our hospital **(A)**, although the ocular fundus was apparently normal in the left eye **(B)**. Two weeks after the second PPV, mutton-fat keratic precipitates **(C,D)** and serous retinal detachment **(E,F)** were found in both eyes. OCT scanning revealed subretinal fluids and elongation of the outer segments **(G,H)**. The UWFA showed delayed perfusion in the early phase, staining of the optic disc, and multi-lake fluorescence accumulation in both eyes **(I–L)**. After 1 week of oral corticosteroids, the patient returned and the subretinal fluids were completely reabsorbed on ultrawide-field photographs **(M,N)** and OCT **(O,P)**.

A 21-year-old woman was referred to the Eye and ENT Hospital of Fudan University for blurred vision in the right eye for about 2 weeks. She had initially presented at her local hospital, and the doctor found a yellowish mass in the temporal peripheral retina accompanied by vitreous hemorrhage. Ocular toxocariasis was suspected and the vitreous sample was collected through the 23-G pars plana vitrectomy (PPV) 5 days later. However, the vitreous antibody to toxocariasis was negative. Her best corrected visual acuities (BCVAs) were 20/40 and 20/20 for the right and left eyes, respectively, on the arrival at our hospital. The ocular examination revealed that the temporal mass was linked to a pair of dilated vessels, however, the persistent vitreous hemorrhage obscured further details, and the left eye was apparently normal (Figures 1A,B). The patient was suspected of retinal capillary hemangioblastoma (RCH), and a second PPV was recommended and performed about 1 month after her arrival at our hospital. During the second PPV, the remaining vitreous hemorrhage was removed, the macular epiretinal membrane was peeled off, and the RCHs were surrounded by laser photocoagulation. The patient came back 2 weeks later and complained of progressively worse vision with distortion in the left eye since the first day after the second PPV. Her BCVAs were 20/40 and 20/50 for the right and left eyes, respectively, at that moment. Mutton-fat keratic precipitates were also found bilaterally (Figures 1C,D). Ultrawide-field photographs showed multifocal serous detachments of the retina in the posterior hole bilaterally (Figures 1E,F). OCT (Spectralis, Heidelberg Engineering Inc., Germany) scans revealed the loss of choroidal vascular architecture, bacillary detachment, and subretinal fluids in both eyes (Figures 1G,H). Ultrawide-field fluorescein angiography (UWFA) (Optos200Tx, Optos Plc., United Kingdom) was ordered based on the suspicion of SO. UWFA revealed hyperfluorescence of the temporal RCHs in the early phase in the right eye (Figure 1I). Delayed choroidal perfusion reflected by the choroidal hypofluorescence and multiple bilateral hyperfluorescent pinpoints in the posterior hole were found in the left eye (Figure 1J). In the late phase, the staining of the optic discs and increased subretinal pooling of fluorescence leaked from the initial pinpoints were also found (Figures 1K,L).

The patient showed no evidence of syphilis or sarcoidosis. Thus, the patient was diagnosed with SO according to the classification criteria for SO proposed by the Standardization of Uveitis Nomenclature Working Group (11). The patient immediately started oral administration of prednisone (1 mg/kg per day). After about 1 week of treatment, the serous retinal detachments resolved bilaterally (Figures 1M,N). However, the borders of the subfoveal choroid were still undetectable on enhanced depth imaging (EDI)-OCT (Figures 1O,P). Her BCVAs improved to 20/20 after 4 months and remained stable with the gradual tapering of oral corticosteroids. On the last visit, after the treatment had been sustained for 1 year, the patient showed no sign of relapse with bilateral BCVAs of 20/20, and prednisone was completely withdrawn.

**Figure 2:**
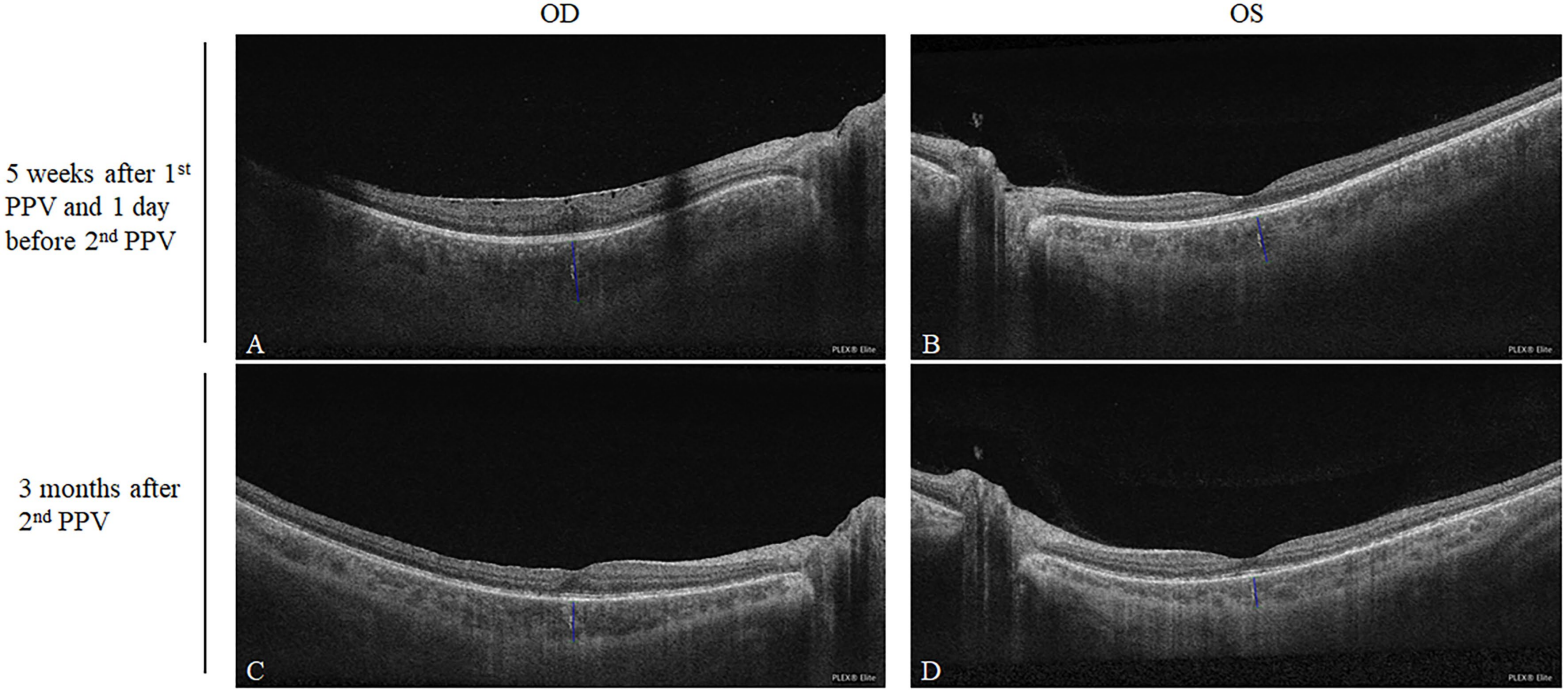
Comparison of the subfoveal choroidal thicknesses after the first PPV and after corticosteroid treatment. Five weeks after the first PPV, the subfoveal choroidal thicknesses of the right and left eyes were 572 and 458 μm, respectively **(A,B)**. Three months after the second PPV being treated with oral corticosteroid, the subfoveal choroidal thicknesses of the right and left eyes decreased to 369 and 290 μm, respectively **(C,D)**.

We searched for any potential signs suggesting SO after the first PPV. OCTA (PLEX Elite 9000, Carl Zeiss Meditec Inc., United States) scanning was performed on the patient as a routine preoperative examination, 1 day before the second PPV, namely, 38 days after the first PPV. B scan through the fovea revealed the macular epiretinal membrane in the right eye and bilateral increased subfoveal choroidal thickness (OD = 572 μm; OS = 458 μm, Figures 2A,B). The examinations were also followed up 3 months after the second PPV and used for self-comparison. In the present case, the choroidal thickness after corticosteroid treatment was smaller than the previous thickness (OD = 369 μm; OS = 290 μm, Figures 2C,D).

**Figure 3:**
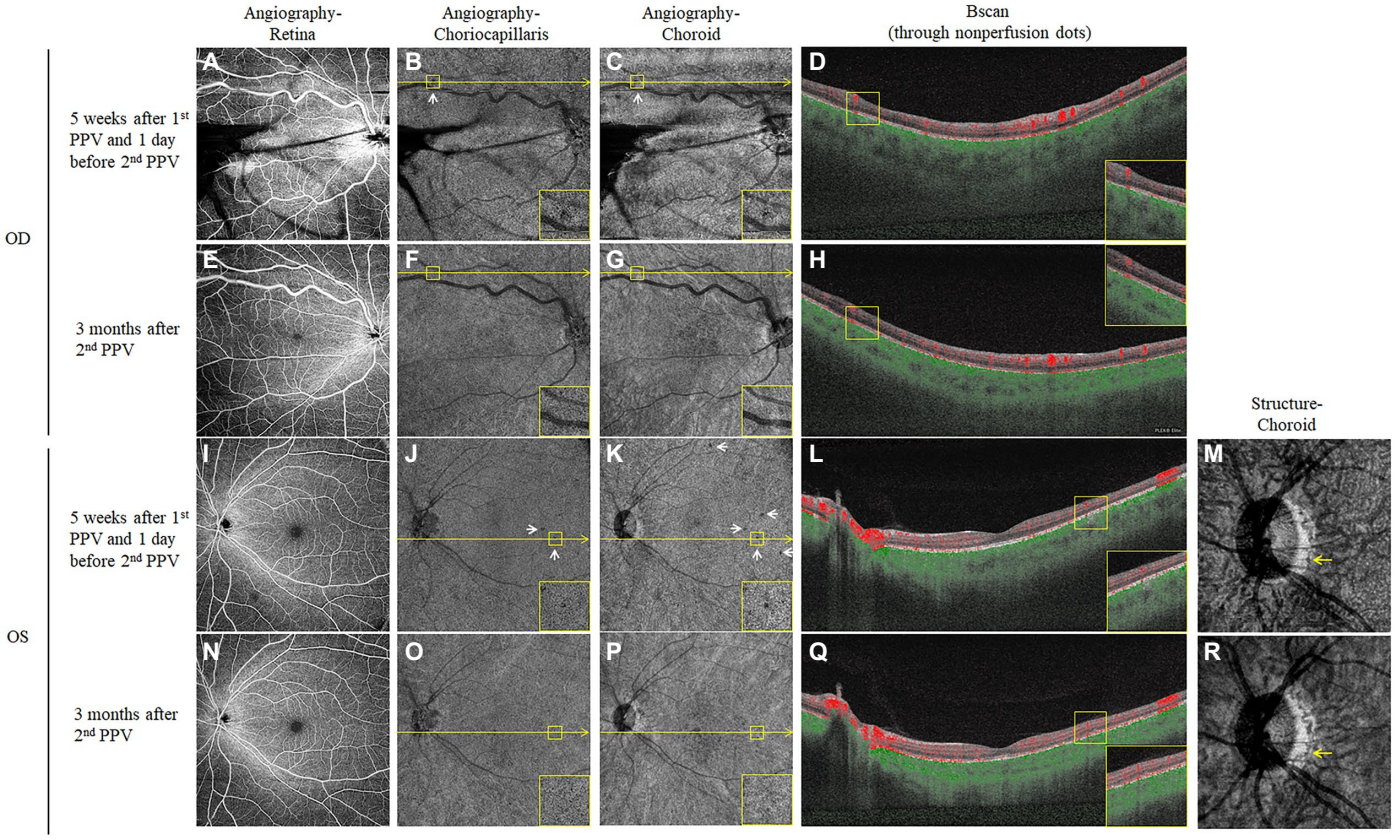
Choroidal vascular changes detected by OCTA after the first PPV and after corticosteroid treatment. Five weeks after the first PPV, the en-face OCTA slabs of the retina were mainly normal for both eyes **(A,I)**, except for the shadowing effects of the vitreous hemorrhage in the right eye. However, the en-face OCTA slabs of both the choriocapillaris **(B,J)** and choroid **(C,K)** showed scattered dark spots (White arrows, yellow boxes in the corners of the panel contain magnified views). The B scan images **(D,L)** were taken along the level of the yellow line through the dark dots, which confirmed flow voids in the choriocapillaris and inner choroid layer (Yellow boxes in the corners of the panel contain magnified views). The choroid en-face structural slab of the left eye also showed an absence of choroidal vessels in the parapapillary area (**M**, yellow arrows). Three months after the second PPV and sustained oral corticosteroid treatment, the OCTA examination was repeated for both eyes. The retinal angiographies were still normal **(E,N)**, the dark dots at the choriocapillaris **(F,O)**, and choroid **(G,P)** levels disappeared at the corresponding locations. The follow-up B scans **(H,Q)** at the positions where the dark dots had been (yellow lines) demonstrated the recovery of the bloodstream signal (Yellow boxes in the corners of the panel contain magnified views). The choroidal vessels also reoccurred on the follow-up choroid en-face structural slab of the left eye (**R**, yellow arrows).

**Figure 4:**
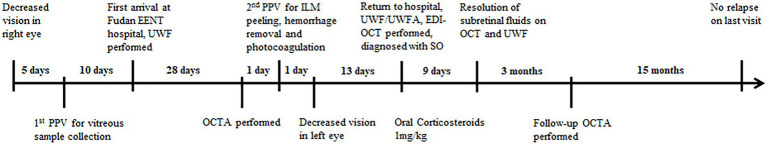
Timeline summarizing the progression of SO. The key events or multimodal imaging examination results in the progression of SO are indicated by short lines along the timeline.

We also turned to the OCTA examination to identify any vascular changes after the first PPV. Five weeks after the first PPV, OCTA images at the level of the retina were generally normal bilaterally, except for the dilated supratemporal vein with tortuosity that was linked to the RCH beyond the scope of the posterior 12 mm × 12 mm (Figures 3A,I). However, at the levels of both the choriocapillaris and choroid, there was a scattering of dots of flow void (Figures 3B,C,J,K, refer to yellow boxes for magnified view). The B scan through these dark spots (Figures 3B,C,J,K, yellow lines) confirmed the absence of the bloodstream signal beneath the RPE-Bruch membrane (Figures 3D,L, refer to yellow boxes for magnified view). After 3 months of oral administration of prednisone, there were still no obvious retinal vascular changes (Figures 3E,N). However, the flow void dots at the levels of both the choriocapillaris and choroid disappeared (Figures 3F,G,O,P, refer to yellow boxes for magnified view). The B scan through the positions corresponding to where the flow voids had been indicated the reappearance of blood stream signal (Figures 3H,Q, refer to yellow boxes for magnified view). Moreover, on the en-face structural images of the choroid, the choroidal vessels in the parapapillary atrophic area reappeared 3 months after the second PPV (Figure 3R, yellow arrow), whereas 5 weeks after the first PPV, the vessel was absent in this position (Figure 3M, yellow arrow). The timeline in Figure 4 summarizes the key events and time intervals during the progression of SO.

Increased SO susceptibility and severity have been reported with HLA-DR4/DQw3, HLA-DRw53 (12), HLA-Cw*03, HLA-DRB1*04, HLA-DQA1*03 (13), HLA-DQB1*04 (14), and HLA-A11 (15). We then performed the whole exome sequencing (WES) of the patient. The germline genetic mutation of VHL (c.500G > A, p.Arg167Gln) confirmed the diagnosis of VHL syndrome. The HLA typing revealed that the two alleles for HLA-A were A11, whereas all the other reported genotypes associated with SO were negative. The detailed typing of encoding genes in the Class I and Class II HLA system is provided in Supplementary Table S1.

## Discussion

There has been an increase in the importance of intraocular surgeries as the first inciting event for SO, and surgical procedures, including vitreoretinal surgeries, also accounted for more than 90% of the last event before SO diagnosis (4). Nonetheless, there has been no reported way to evaluate that in whom inflammation has been started by an inciting event but still without typical manifestations of SO. In this report, we describe the presymptomatic choroidal changes at the early stage of SO, which suggests the started inflammatory response and could be used to inform surgical decision-making and even guide interventions in SO.

Classically, SO is considered to affect the choroid initially and mainly. Moreover, the involvement of choriocapillaris has also been confirmed histopathologically (16). In the acute phase of SO, the choroidal thickness is increased on EDI-OCT because of diffuse infiltration of inflammatory cells in choroidal stroma (17), and the hypocyanescent spots on indocyanine green angiography (ICGA) have been interpreted as focal cellular infiltration into the choroid or formation of Dalen-Fuchs nodules (18). However, because most observations of patients with SO have been performed after its typical manifestations occur, evidence of choroid and choriocapillaris involvement at an early stage has been missing. In this case, we found that first, the subfoveal choroidal thickness increased before the manifestations of SO. The choroidal thickness varies in the population, depending on the age and refractive errors, and it may also increase reactively and temporarily in a postoperative eye. However, the involvement of the contralateral eye without surgeries and a significant decrease in the choroidal thickness after therapy strongly supported that the increased thickness resulted from the unnoticed SO at the beginning. Furthermore, flow void dots on the OCTA choroid and choriocapillaris en-face slabs were identified. In Vogt–Koyanagi–Harada (VKH) disease, another uveitis resembling SO, areas of flow void on both choriocapillaris and choroid en-face slabs in OCTA correspond well to the hypocyanescent lesions on ICGA (19, 20). Thus, the flow voids in the present case may also suggest the underlying inflammatory foci or granulomas and subsequent ischemic changes. To the best of our knowledge, these findings are the first to indicate the inflammatory involvement of the choroid and choriocapillaris in both inciting and sympathetic eyes before the manifestations of SO.

Although the autoimmune responses in SO are initiated by the first inciting event, subsequent surgical injuries may release more self-antigens, resulting in augmentation of responses and exacerbation of SO (21). Nonetheless, it is difficult to recognize the patients with presymptomatic SO after the first trigger event, in whom SO would be aggravated by another surgical intervention. In this report, the choroid thickening and flow void dots of the choroid and choriocapillaris after the first PPV were found afterward by retrospective comparison, which supports already existing SO, albeit at the presymptomatic and unnoticed stage. Such findings may warrant close follow-up and caution toward additional surgical interventions. In summary, our findings support a pre-surgery bilateral examination with OCTA, which should be sufficiently sensitive to recognize the unnoticed SO. However, our observation was limited to one case owing to the rarity of SO; thus, a larger observational study is needed to confirm the presymptomatic stage before SO manifests typically and to investigate whether any intervention is helpful.

The overall visual prognosis after treatment in Chinese patients with SO was worse than that in patients with VKH disease (22). In the present case, SO quickly responded well to corticosteroid treatment without additional immunosuppressive agents. There were no flare-ups throughout the follow-up, and visual acuity returned to normal and remained stable. Genetic analysis revealed double HLA-11 alleles, which may predispose the present case to develop SO according to the study of Reynard M et al. (15). However, besides the HLA system, the effects of other non-HLA gene variation on the progression and prognosis of SO remain largely elusive. In our case, SO developed on the background of VHL missense mutation (c.500G > A). The roles of VHL deficiency in the T cell-mediated response are not always consistent. VHL deficiency facilitates Th1dominant responses (23), but halts the differentiation of follicular helper T cells (24) and Th17 cells, and thus confers protection against experimental autoimmune encephalomyelitis (8). Most recently, VHL deficiency in CD4 + T cells also accounts for their impaired responses and susceptibility to tuberculosis infection (25). Although the prompt initiation of treatment played a role in the relatively benign SO progression and favorable outcomes here, VHL mutation is likely to regulate the duration, and magnitude of the Th17-mediated autoimmunity, and even the response to corticosteroid treatment. Since definite evidence is still lacking, further laboratory investigations are needed to elucidate the effects of VHL deficiency on the progression of SO.

## Conclusion

In conclusion, we observed the progression of SO after multiple PPVs on the background of VHL syndrome-associated RCHs. The increased choroid thickness and discrete flow void dots at the presymptomatic stage of SO suggested the early involvement of the choroid. Based on our observations, the screening of bilateral eyes by OCTA is recommended for patients with antecedent inciting events, especially before additional surgical interventions. Patients with red flag signs should be informed of the potential risk that ensuing surgeries could accelerate and exacerbate the inflammation, and close postsurgical follow-up is necessary.

## Data availability statement

The datasets generated during and/or analyzed during the current study are included in the article and Supplementary material, further information was available from the corresponding author on a reasonable request.

## Ethics statement

The studies involving human participants were reviewed and approved by the IRB/Ethics Committee of Eye and ENT Hospital of Fudan University. The patients/participants provided their written informed consent to participate in this study. Written informed consent was obtained from the individual(s) for the publication of any potentially identifiable images or data included in this article.

## Author contributions

GX obtained funding for the study and supervised the report. XZ, FG, ZS, and XD performed the clinical examination and interpreted the clinical data. FG performed the genetic analysis. XZ and FG drafted and revised the manuscript. All authors contributed to the article and approved the submitted version.

## Funding

This study was supported by the Clinical Research Plan of SHDC (SHDC2020CR2041B).

## Conflict of interest

The authors declare that the research was conducted in the absence of any commercial or financial relationships that could be construed as a potential conflict of interest.

## Publisher’s note

All claims expressed in this article are solely those of the authors and do not necessarily represent those of their affiliated organizations, or those of the publisher, the editors and the reviewers. Any product that may be evaluated in this article, or claim that may be made by its manufacturer, is not guaranteed or endorsed by the publisher.
